# The influence of 17β-estradiol plus norethisterone acetate treatment on markers of glucose and insulin metabolism in women: a systematic review and meta-analysis of randomized controlled trials

**DOI:** 10.3389/fendo.2023.1137406

**Published:** 2023-05-17

**Authors:** Weijuan Cui, Ling Zhao

**Affiliations:** Department of Endocrinology, the First People’s Hospital of Linping District, Hangzhou, China

**Keywords:** glucose markers, insulin, insulin resistance, HbA1c, 17β-estradiol, norethisterone acetate

## Abstract

**Objective:**

Despite the fact that some evidence suggests that the administration of 17β-estradiol plus norethisterone acetate influences glucose and insulin metabolism in women, these findings are still contradictory. Thus, we aimed to examine the impact of the co-administration of 17β-estradiol and norethisterone acetate on glycated haemoglobin (HbA1c), fasting glucose, insulin and C-peptide concentrations in females by means of a systematic review and meta-analysis of randomized controlled trials (RCTs).

**Methods:**

We searched four databases (PubMed/MEDLINE, Scopus, Embase, and Web of Science) using specific keywords and word combinations. The random-effects model (DerSimonian and Laird model) was employed to compute the weighted mean difference (WMD) and 95% confidence intervals (CIs) for the variations from baseline of HbA1c, fasting glucose, insulin, and C-peptide concentrations.

**Results:**

In total, 14 RCTs were entered into the quantitative synthesis. The combined administration of 17β-estradiol and norethisterone acetate decreased HbA1c (WMD: -0.65%, 95% CI: -1.15 to -0.15; P=0.011), fasting glucose (WMD: -11.05 mg/dL, 95% CI: -16.6 to -5.5; P<0.001) and insulin (WMD: -1.35 mIU/L, 95% CI: -2.20 to -0.50; P=0.001) levels. C-peptide concentrations’ declined only in females diagnosed with overweight/obesity or diabetes.

**Conclusion:**

Evidence to date points out that the administration of 17β-estradiol and norethisterone acetate has a positive impact on glucose metabolism in women by reducing fasting glucose, HbA1c, and insulin values. Future studies need to confirm the potential benefits of this drug combination in the prevention and/or management of cardiometabolic disorders.

## Introduction

There is a gradual decline in estrogen levels and in their cardioprotective effects in women as they approach the menopausal age (1). As females attain menopause, they are prone to develop several menopause-related symptoms or diseases, e.g., hot flashes, cardiovascular disorders, dementia, osteoporosis, sexual, and urogenital dysfunctions (2–4). To control or alleviate menopause-related symptomatology and to improve quality of life, women of postmenopausal age will require hormone replacement therapy (HRT) (2, 5). However, available evidence suggests that the administration of HRT can influence the metabolism of glucose and insulin (6–8).

An increase in insulin resistance and deterioration in glucose tolerance is known to occur with advancing age, although the effect of menopause remains controversial (9). Moreover, a lack of estrogen tends to promote an accumulation of abdominal fat, which is a major cardiovascular risk factor and one of the criteria for the diagnosis of metabolic syndrome (10). Estrogen may have beneficial effects on carbohydrate metabolism while the effects of progestogen may be deleterious; however, results may differ depending on the type of progestogen used (11). Due to conflicting results from previously conducted studies, the actions of 17β-estradiol plus norethisterone acetate on glucose homeostasis in women remain controversial (6–8, 12–22). Thus, we conducted a systematic review and meta-analysis of randomized controlled trials (RCTs) to investigate the effects of 17β-estradiol plus norethisterone acetate treatment on fasting glucose, fasting insulin, glycated haemoglobin (HbA1c) and C-peptide in women.

## Materials and methods

This meta-analysis was accomplished in agreement with the Preferred Reporting Items for Systematic Reviews and Meta-Analyses (PRISMA) statements (23).

### Search strategy

The PubMed/MEDLINE, Scopus, Embase, and Web of Science databases were systemically searched without language restrictions to identify papers indexed before June 14^th^, 2022. In addition, to identify other papers that could have been missed in the previous step, we checked the lists of references of any relevant review and/or eligible study. The MeSH (Medical subject heading) and non-MeSH search keywords used: (((“Glycated Hemoglobin A”[Mesh] OR “Blood Glucose”[Mesh] OR “Hyperglycemia”[Mesh] OR “Insulin Resistance”[Mesh] OR “Hyperinsulinism”[Mesh] OR “Hyperinsulinism”[Mesh] OR “Diabetes Mellitus”[Mesh] OR “Glycosylated Hemoglobin”[Title/Abstract] OR “HbA1”[Title/Abstract] OR “Hb A1c”[Title/Abstract] OR “Glycohemoglobin A”[Title/Abstract] OR “Glycated Hemoglobin”[Title/Abstract] OR “Blood Glucose”[Title/Abstract] OR “Blood Sugar”[Title/Abstract] OR Hyperglycemia[Title/Abstract] OR Glycemia[Title/Abstract] OR HOMA*[Title/Abstract] OR “homeostatic model for insulin resistance”[Title/Abstract] OR “fasting blood insulin”[Title/Abstract] OR FBI[Title/Abstract] OR FBG[Title/Abstract] OR FBS[Title/Abstract] OR “Insulin Resistance”[Title/Abstract] OR “Insulin Sensitivity”[Title/Abstract] OR “Hyperinsulinemia”[Title/Abstract] OR Hyperinsulinism[Title/Abstract] OR Insulin*[Title/Abstract] OR Hyperinsulin*[Title/Abstract] OR “Oral Glucose Tolerance Test”[Title/Abstract] OR OGTT[Title/Abstract] OR “Diabetes Mellitus”[Title/Abstract]))) AND (((estrogen [Mesh] OR estrogen [tiab] OR estrogen replacement therapy [Mesh] OR estrogen replacement therapy [tiab] OR hormone replacement therapy [Mesh] OR hormone replacement therapy [tiab] OR estradiol [Mesh] OR estradiol [tiab] OR progestin therapy [Mesh] OR progestin therapy [tiab] OR progestin [Mesh] OR progestin [tiab] OR *progesterone [Mesh] OR *progesterone [tiab] OR HRT [Mesh] OR HRT [tiab] OR tibolone [Mesh] OR tibolone [tiab] OR norethisterone [Mesh] OR norethisterone [tiab] OR norethindrone [Mesh] OR norethindrone [tiab] OR medrogestone [Mesh] OR medrogestone [tiab]))) AND (((((“Clinical Trials as Topic”[Mesh] OR “Cross-Over Studies”[Mesh] OR “Double-Blind Method”[Mesh] OR “Single-Blind Method”[Mesh] OR “Random Allocation”[Mesh] OR RCT[Title/Abstract] OR “Clinical Trial” [Publication Type] OR “Controlled Clinical Trials as Topic”[Mesh] OR “Intervention Studies”[Title/Abstract] OR “intervention”[Title/Abstract] OR Trial[Title/Abstract] OR “controlled trial”[Title/Abstract] OR “randomized”[Title/Abstract] OR “randomised”[Title/Abstract] OR “random”[Title/Abstract] OR “randomly”[Title/Abstract] OR “placebo”[Title/Abstract] OR “assignment”[Title/Abstract]))))).

### Eligibility criteria

To select the eligible RCTs, we used the PICO framework (P: women; I: 17β-estradiol plus norethisterone acetate in oral form; C: a group of women who did not receive 17β-estradiol plus norethisterone acetate and only received standard of care therapy or any other medication as control/placebo; O: mean and standard deviation (SD) for HbA1c, fasting glucose, insulin, and c-peptide. All papers other than original research publications (letter to the editor, correspondence, reviews), case series or case reports, studies on pregnant females and on participants below 18 years of age were excluded.

### Data extraction

Two researchers accomplished the data extraction independently. In addition, the disagreements and discrepancies were resolved via consultation with the head author. The following data regarding the RCTs were extracted: (1) general information (i.e., publication year, title, authors, and country); (2) methodological information (i.e., treatment allocation, trial period duration, intervention description, and study design),; (3) participant-related data (i.e., sample size, group, age, sex, baseline characteristics and participant demographics); and (4) result-related data (i.e., mean and SD of HbA1c, fasting glucose, insulin, and c-peptide).

### Quality assessment

The quality of the RCTs was measured independently by two investigators using the Cochrane collaboration’s tool (24). This instrument evaluates the next parameters: incomplete outcome data, allocation concealment, sequence generation, blinding of participants and personnel, blinding of outcome assessment, selective outcome reporting, and other sources of bias.

### Statistical analysis

The random-effects model (DerSimonian and Laird model) was used to compute the weighted mean difference (WMD) and 95% confidence intervals (CIs) for the variations from baseline of HbA1c, fasting glucose, insulin, and C-peptide values. We measured the heterogeneity by employing the I^2^ test. Values of <25%, 26–50%, and >50% indicated low, moderate and high degrees of heterogeneity, respectively.

Moreover, we evaluated the risk of publication bias by the Egger’s tests (quantitative) and funnel plot analysis (qualitative) (25). When significant publication bias was detected among the RCTs, the trim-and-fill test was applied to estimate the impact of unpublished papers (26). In order to ascertain the potential source of heterogeneity, stratified analyses were executed based on the following parameters: the length of the intervention, the participants’ body mass index (BMI) at baseline, the subjects’ health status, and the participants’ mean age. We employed a sensitivity analysis test to calculate the vigor of the overall results by exclusion of each comparison one-by-one and computing the final result. The statistical analysis of the current investigation was run using Stata software version 14 (StataCorp, TX, USA).

## Results

### Study selection

**Figure 1:**
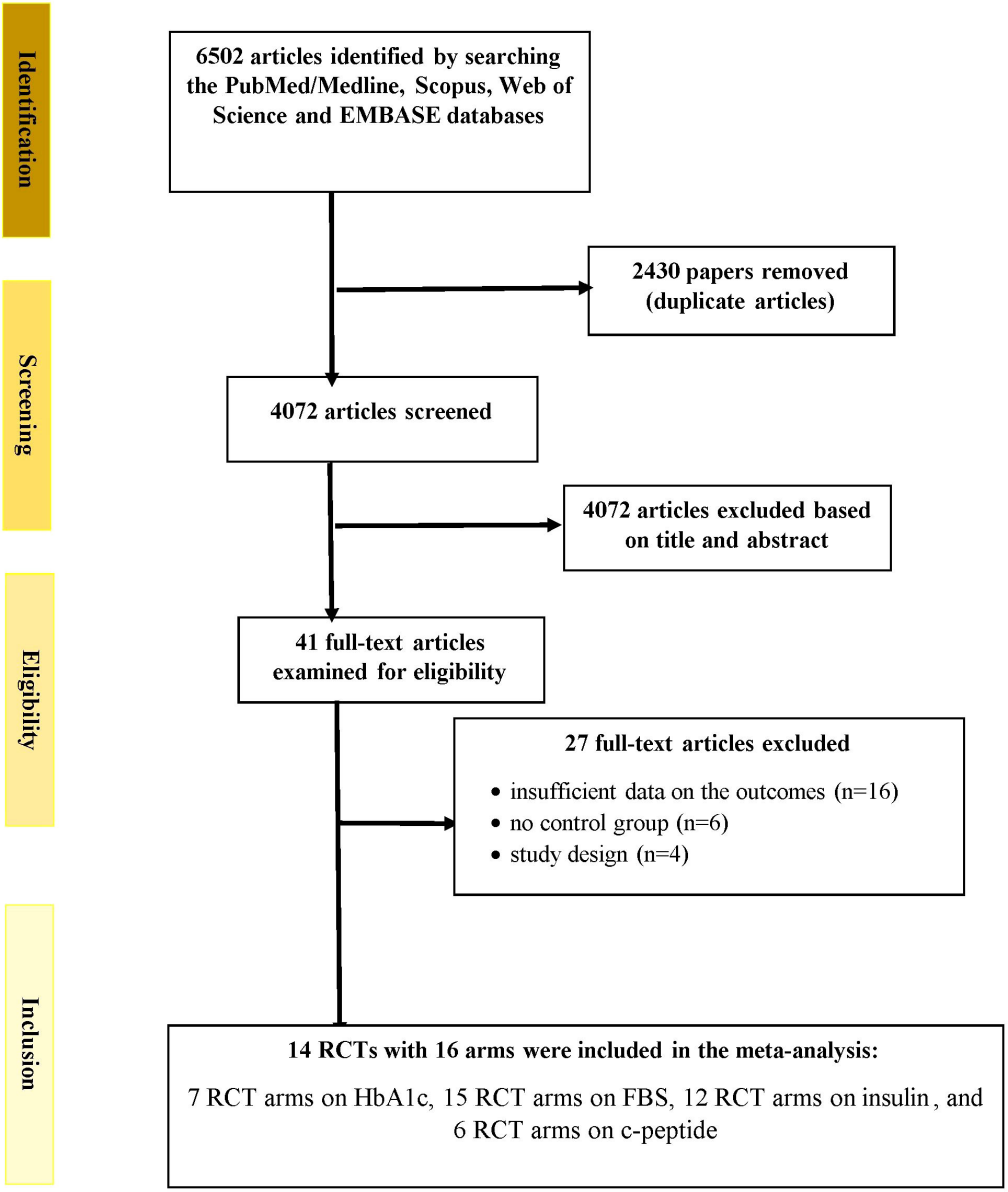
Flowchart depicting the study selection and inclusion processes for the current meta-analysis. RCTs, randomized controlled trials; HbA1c, glycated hemoglobin; FBS, fasting blood glucose.

**Figure 1** depicts the study selection method. Two researchers accomplished the study selection independently. In addition, the disagreements and discrepancies were resolved via consultation with the head author. A total of 6502 publications was found when the databases were searched. An overall of 2430 records were retrieved after the removal of duplicates. During the screening of titles and abstracts step, 4072 publications were excluded. In the next stage, of the 41 full-texts assessed, 16 papers were eliminated due to unavailability of the results, 4 articles were excluded because they lacked an RCT design, and 6 papers were excluded because they lacked a control group. Finally, 14 RCTs with 16 arms were included in the meta-analysis: 7 RCT arms on HbA1c, 15 RCT arms on fasting glucose, 12 RCT arms on insulin, and 6 RCT arms on c-peptide (13–22, 27–31).

### Characteristics of the included studies

**Table 1** depicts the characteristics of the eligible publications. These RCTs were conducted in the United Kingdom, Brazil, Sweden, Turkey, Finland, Sweden, New Zealand, Italy, Germany, and Singapore. All of the 17β-estradiol plus norethisterone acetate doses were given orally, although the dosage varied between the eligible publications, ranging from 700 µg/day to 1 mg/day for norethisterone acetate and 1 mg/day to 2 mg/day for 17β-estradiol. All manuscripts were published between 1992 and 2013. The recruited subjects were healthy women or females diagnosed with type 2 diabetes mellitus (T2DM), overweight or obesity. The period of the intervention varied from 12 weeks to 2 years. The mean age of the participants varied from 25.4 years to 62.2 years. **Supplementary Table 1** delineates the quality assessment of the included RCTs.

**Table 1:** Characteristics of the eligible studies.

Author	Publications years	Country	Population	Participants’ age (years)	Sample size: intervention/placebo	Duration	Baseline BMI(kg/m^2)^	Outcome	17β-estradiol (estradiol) plus norethisterone acetate dose
Manassiev, N.	2013	UK	postmenopausal women	62	31/30	2 years	23.5	Insulin, C-peptide	E2 2 mg/NETA 1 mg daily
Fernandes, C. E.	2008	Brazil	postmenopausal women	52	28/24	6 months	27	FBS, Insulin	2 mg E2/1 mg NETA
Kernohan, A. F.	2007	UK	postmenopausal women with T2DM	62	15/15	3 months	34	FBS, HbA1c	17β-estradiol (1 mg) and norethisterone (0.5 mg)
Thunell, L.	2006	Sweden	postmenopausal women with T2DM	62	31/31	6 months	29	FBS, HbA1c, Insulin, C-peptide	2 mg estradiol and 1 mg norethisterone
Osmanagaoglu, M. A.	2005	Turkey	postmenopausal women with overweight/obesity	50	90/88	6 months	28	FBS, Insulin	2 mg of E2 plus 1 mg of norethisterone acetate (E2/NETA)
McKenzie, J.	2003	Finland	postmenopausal women with T2DM	60	19/22	6 months	30.5	FBS, HbA1c, C-peptide	2 mg of E2 plus 1 mg of norethisterone acetate (E2/NETA)
Samsioe, G.	2002	Sweden	healthy postmenopausal women	55	33/17	12 months	25.2	FBS, Insulin	1 mg estradiol and 0.5 mg norethisterone
Samsioe, G.	2002	Sweden	healthy postmenopausal women	56	35/17	12 months	25.5	FBS, Insulin	1 mg/day E2 plus 0.25 mg/day NETA
Walker, R. J.	2001	New Zealand.	healthy postmenopausal women	46	15/15	6 months	28	FBS, Insulin	1 mg/day E2 plus 0.5 mg/day NETA
Ventura, P.	2001	Italy	postmenopausal women	55	11/11	6 months	24	FBS	2 mg of 17β-estradiol for days 1-12, 2 mg of 17β-estradiol and 1 mg of norethisterone acetate
Darko, D. A.	2001	UK	menopausal women with T2DM	nr	11/13	12 weeks	28	FBS, HbA1c, Insulin	2 mg of estradiol plus 1mg of norethisterone acetate
Seed, M.	2000	UK	postmenopausal women	58	45/83	6 months	26.5	FBS, Insulin	17β-estradiol 2 mg and norethisterone 1 mg
Kimmerle, R.(a)	1999	Germany	postmenopausal women	55	18/9	3 months	25	FBS, HbA1c Insulin, C-peptide	estradiol 2 mg and norethisterone 700ug/daily
Kimmerle, R.(b)	1999	Germany	postmenopausal women	57	18/9	3 months	25	FBS, HbA1c Insulin, C-peptide	2 mg/day E2 plus1 mg/day NETA
Andersson, B.	1997	Sweden	postmenopausal women with noninsulin-dependent T2DM	59	25/25	3 months	30	FBS, HbA1c Insulin, C-peptide	1 mg/day E2 plus 0.5 mg/day NETA
Loke, D. F. M.	1992	Singapore	women	25	29/23	12 months	21	FBS	2 mg/day E2 plus1 mg/day NETA

mg/d, milligrams per day.WMD, weighted mean difference. CI, confidence interval. HbA1c, glycated hemoglobin. FBS, fasting blood glucose. BMI, body mass index. UK, United Kingdom. T2DM, type 2 diabetes mellitus. E2, 17β-estradiol (estradiol). NETA, norethisterone acetate.

### Findings from the meta-analysis

#### Effects of 17β-estradiol plus norethisterone acetate on HbA1c levels

**Figure 2:**
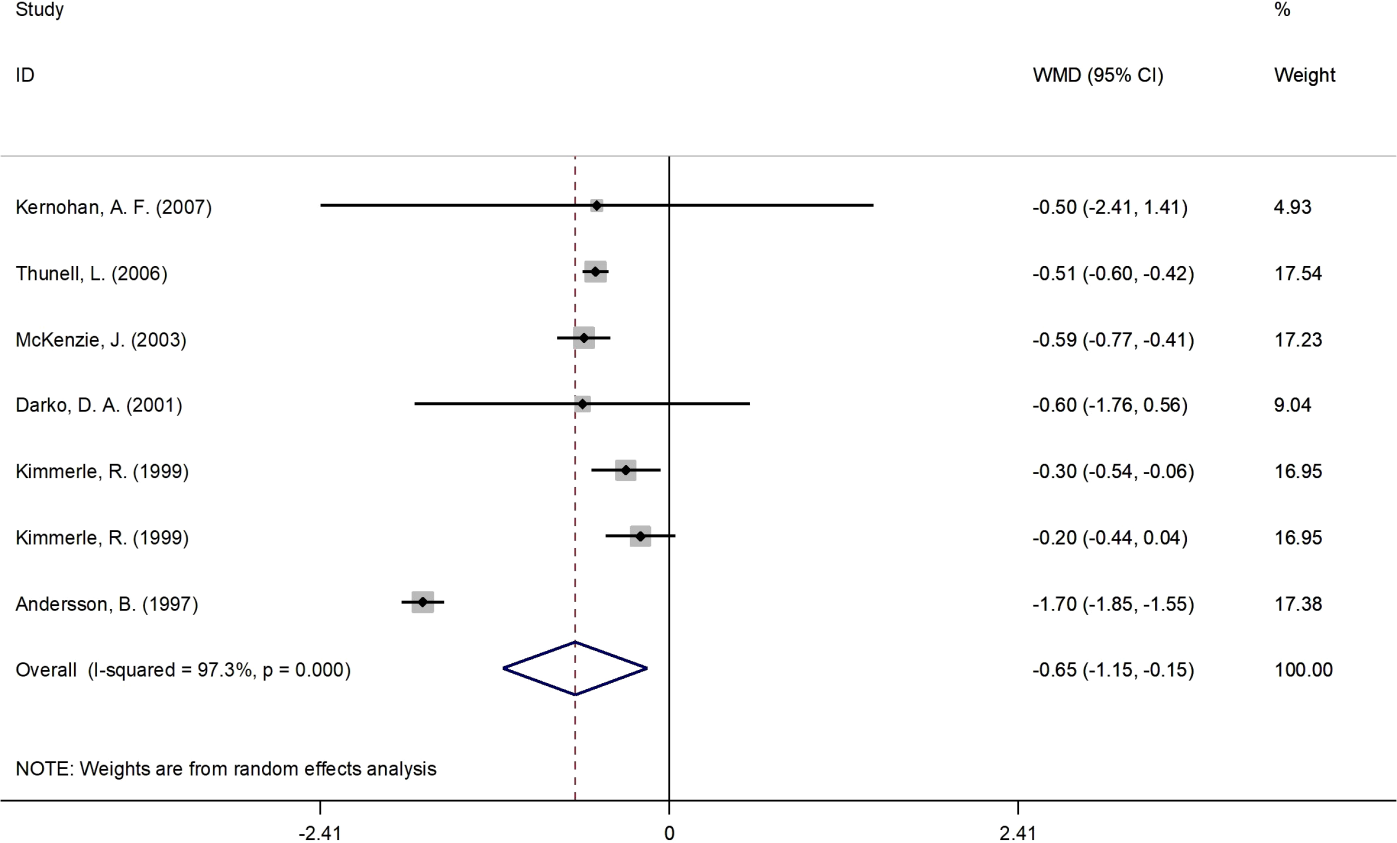
Forest plot of RCTs investigating the effects of 17β-estradiol plus norethisterone acetate on HbA1c. RCTs, randomized controlled trials; HbAlc, glycated hemoglobin; WMD, weighted mean difference; CL, confidence interval.

**Figure 2** presents the forest plot for HbA1c concentrations. A total of 7 RCT arms (sample size = 261 subjects; 17β-estradiol plus norethisterone acetate group = 137 subjects, placebo group = 124 subjects) evaluated the impact of 17β-estradiol plus norethisterone acetate administration on HbA1c levels in postmenopausal females. Women who were prescribed 17β-estradiol plus norethisterone acetate experienced significant reductions in HbA1c concentrations (WMD: -0.65%, 95% CI: -1.15 to -0.15; P=0.011) versus the placebo group. However, a significant heterogeneity was noticed among the trials (I^2 = ^97%, P<0.001). Moreover, a significant decrease in HbA1c values was observed in postmenopausal women aged ≥60 years (WMD: -0.50%, 95% CI: -0.60 to -0.45, P<0.001) versus <60 years (WMD: -0.73%, 95% CI: -1.80 to 0.33, P=0.17). In addition, a notable decline in HbA1c levels was demonstrated in postmenopausal women with a BMI of ≥30 kg/m^2^ (WMD: -1.05%, 95% CI: -2.05 to -0.05, P=0.03) versus <30 kg/m^2^ (WMD: -0.4%, 95% CI: -0.6 to -0.2, P<0.001). The subgroup analyses depicted significant reductions in HbA1c values in the RCTs with a duration of ≥6 months (WMD: -0.50%, 95% CI: -0.60 to -0.45, P<0.001) versus <6 months (WMD: -0.70%, 95% CI: -1.6 to 0.2, P=0.129). Moreover, a pronounced decrease in HbA1c concentrations was observed in postmenopausal women with T2DM (WMD: -0.85%, 95% CI: -1.50 to -0.2, P=0.01) versus healthy postmenopausal women (WMD: -0.25%, 95% CI: -0.40 to -0.08, P=0.004) (**Supplementary Figure 1**).

#### Effects of 17β-estradiol plus norethisterone acetate on fasting glucose levels

**Figure 3:**
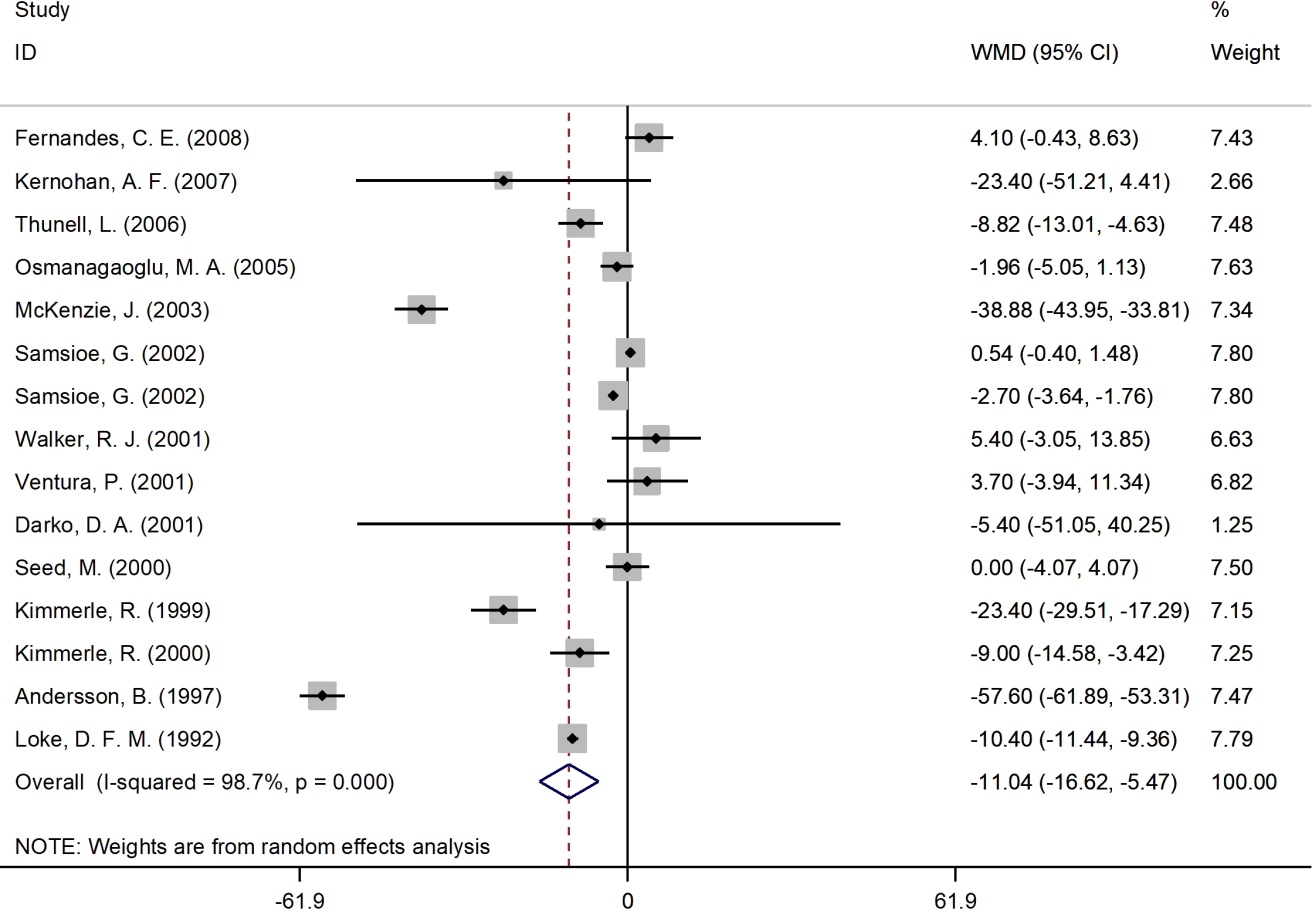
Forest plot of RCTs investigating the effects of 17β-estradiol plus norethisterone acetate administration on FBS. RCTs, randomized controlled trials. FBS, fasting blood glucose; WMD, weighted mean difference; CI, confidence interval.

**Figure 3** presents the forest plot for fasting glucose concentrations. A total of 15 RCT arms (sample size = 825 subjects; 17β-estradiol plus norethisterone acetate group = 423 subjects; placebo group = 402 subjects) assessed the effect of 17β-estradiol plus norethisterone acetate administration on fasting glucose levels in postmenopausal female. There was a notable decrease in fasting glucose concentrations in women who received 17β-estradiol plus norethisterone acetate (WMD: -11.05 mg/dL, 95% CI: -16.6 to -5.5; P<0.001) versus placebo. However, a significant heterogeneity was noticed among the trials (I^2 = ^98%, P<0.001). fasting glucose levels notably decreased in postmenopausal women aged <60 years (WMD: -8.45 mg/dL, 95% CI: -14.25 to -2.60, P<0.001) versus ≥60 years (WMD: -23.70 mg/dL, 95% CI: -48.5 to 1.05, P=0.06). In addition, a significant reduction in fasting glucose levels was detected in postmenopausal women with a BMI of ≥25 kg/m^2^ (WMD: -12.30 mg/dL, 95% CI: -19.25 to -5.40, P<0.001) versus <25 kg/m^2^ (WMD: -3.90 mg/dL, 95% CI: -17.65 to 9.90, P=0.58). The subgroup analyses also revealed a significant decrease in fasting glucose values in the RCTs with a duration of ≥6 months (WMD: -5.10 mg/dL, 95% CI: -9.55 to -0.70, P=0.02) versus <6 months (WMD: -25.60 mg/dL, 95% CI: -50.75 to -0.50, P=0.046). Moreover, a significant decrease in fasting glucose concentrations was observed in women with T2DM (WMD: -29.20 mg/dL, 95% CI: -53.50 to -4.90, P=0.01) versus healthy women (WMD: -3.50 mg/dL, 95% CI: -7.30 to 0.25, P=0.06) (**Supplementary Figure 1**).

#### Effects of 17β-estradiol plus norethisterone acetate on insulin levels

**Figure 4:**
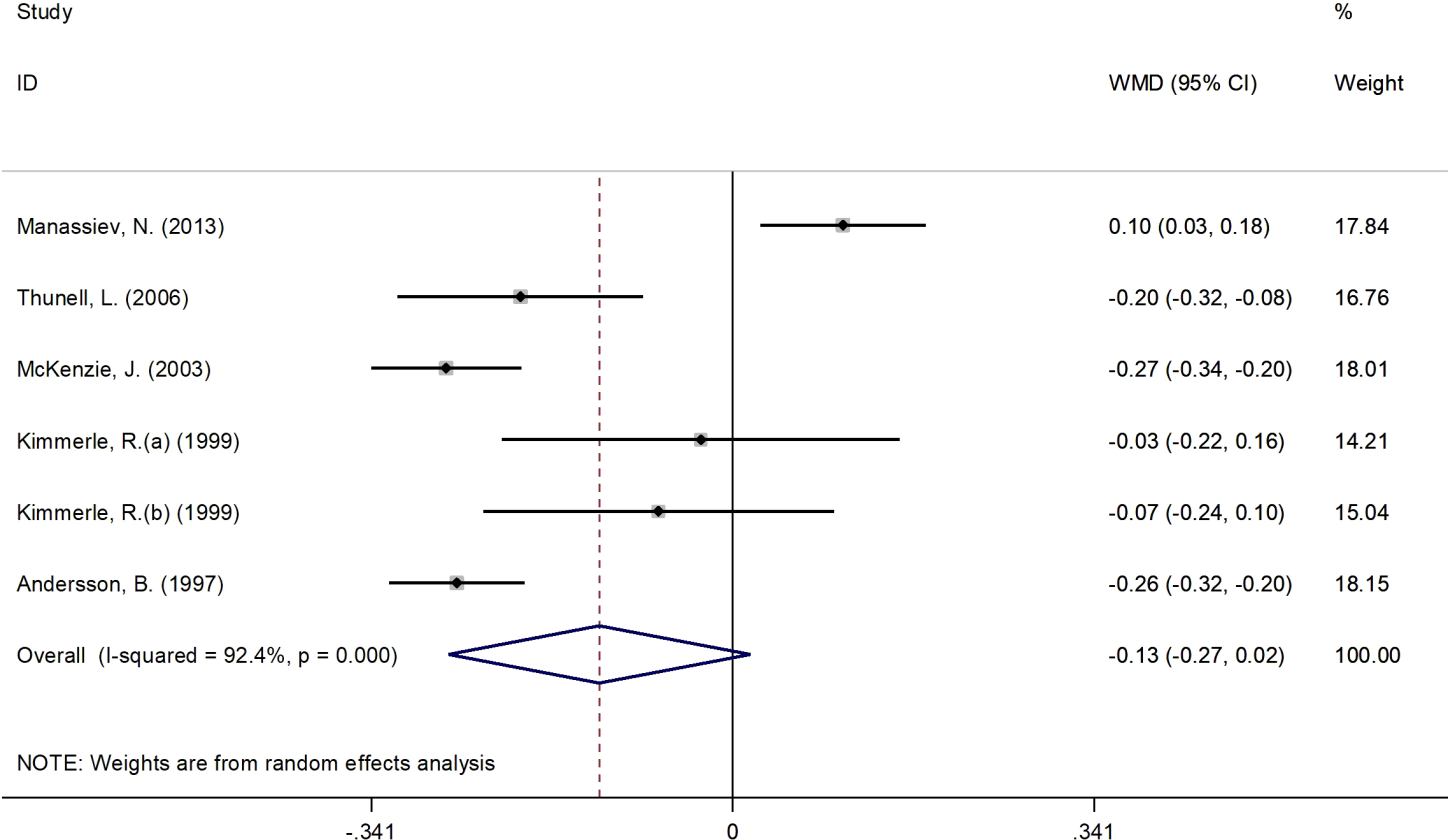
Forest plot of RCTs investigating the effects of 17β-estradiol plus norethisterone acetate on insulin levels. RCT, randomized controlled trials; WMD, weighted mean difference; Cl, confidence interval.

**Figure 4** presents the forest plot for insulin concentrations. A total of 12 RCT arms with (sample size = 810 subjects; 17β-estradiol plus norethisterone acetate group = 404 subjects; placebo = 406 subjects) investigated the action of 17β-estradiol plus norethisterone acetate administration on insulin levels in females. Following the intake of 17β-estradiol plus norethisterone acetate, insulin concentrations decreased (WMD: -1.35 mIU/L, 95% CI: -2.20 to -0.50; P=0.001), with a moderate heterogeneity among the trials (I^2 = ^38%, P=0.08). Moreover, a significant decline in insulin levels was noted in women aged ≥60 years (WMD: -1.60 mIU/L, 95% CI: -2.45 to -0.80, P<0.001) versus <60 years (WMD: -1.10 mIU/L, 95% CI: -2.25 to 0.06, P=0.06). In addition, a pronounced reduction in insulin concentrations was experienced by women with a BMI of ≥25 kg/m^2^ (WMD: -1.4 mIU/L, 95% CI: -2.20 to -0.50, P=0.002) versus <25 kg/m^2^ (WMD: 1.1 mIU/L, 95% CI: -6.20 to 8.30, P=0.77). The subgroup analyses discovered a significant decrease in insulin values in the RCTs with a duration of ≥6 months (WMD: -1.20 mIU/L, 95% CI: -2.05 to -0.40, P=0.005) versus <6 months (WMD: -1.40 mIU/L, 95% CI: -3.75 to 0.90, P=0.23). Moreover, a notable reduction in insulin concentrations was observed in women with T2DM (WMD: -2.20 mIU/L, 95% CI: -3.40 to -1.10, P<0.001) versus healthy women (WMD: -0.80 mIU/L, 95% CI: -1.80 to 0.20, P=0.12) (**Supplementary Figure 1**).

#### Effects of 17β-estradiol plus norethisterone acetate on C-peptide levels

**Figure 5:**
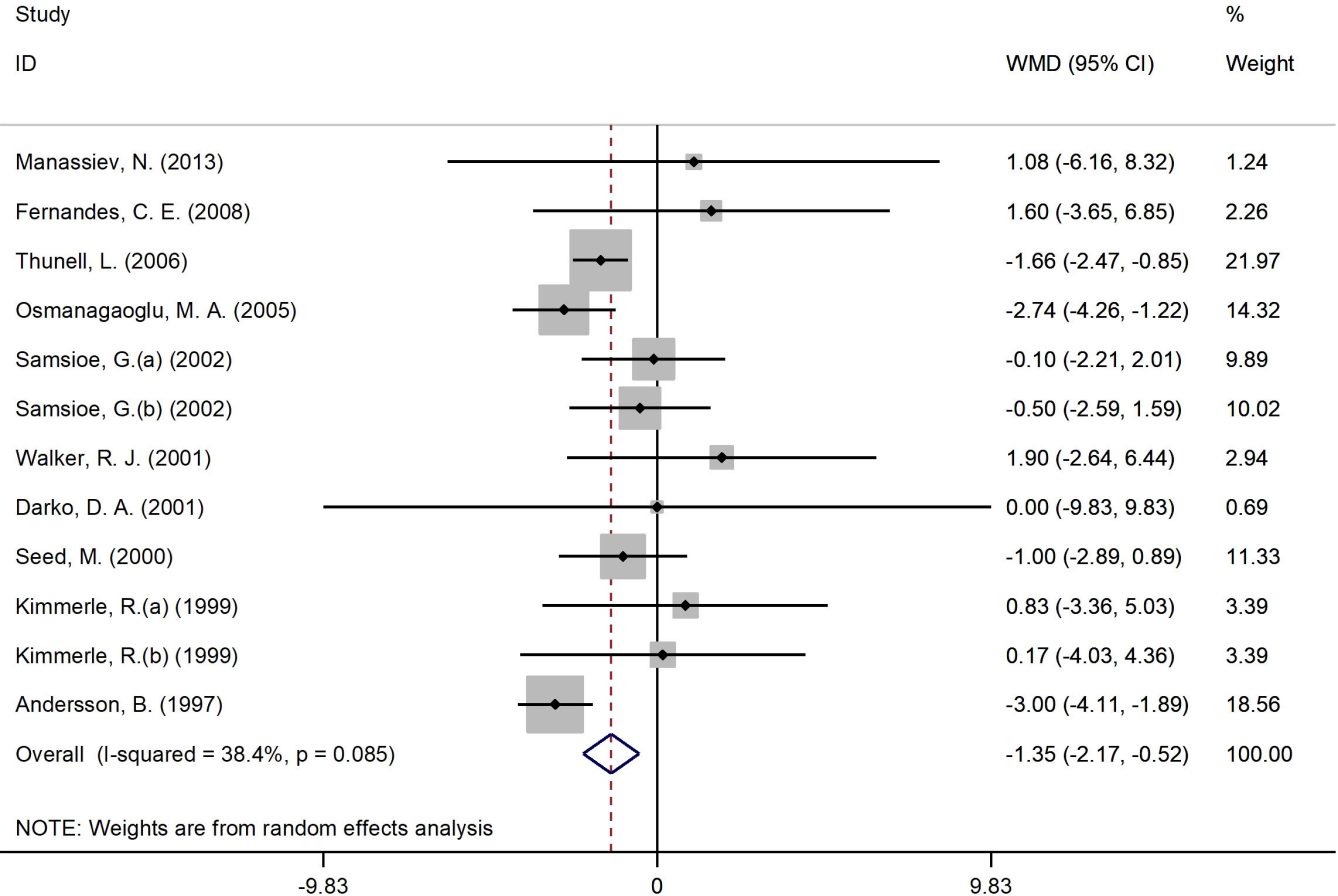
Forest plot of RCTs investigating the effects of 17β-estradiol plus eorethisterone acetate administration on C-peptide levels. RCTs, and contriled trials; WMD, weighted mean difference; Ct, confidence umurval.

**Figure 5** presents the forest plot for C-peptide concentrations. A total of 6 RCT arms (sample size = 268 subjects; 17β-estradiol plus norethisterone acetate group = 142 subjects; placebo = 126 subjects) evaluated the impact of 17β-estradiol plus norethisterone acetate administration on C-peptide levels in females. C-peptide concentrations did not change following the use of 17β-estradiol plus norethisterone acetate (WMD: -0.12 nmol/L, 95% CI: -0.30 to 0.02; P=0.08) versus placebo, with significant heterogeneity among the trials (I^2 = ^92%, P<0.001). However, a notable reduction in C-peptide levels was detected in women with a BMI of ≥25 kg/m^2^ (WMD: -0.20 nmol/L, 95% CI: -0.30 to -0.15, P<0.001) versus <25 kg/m^2^ (WMD: 0.10 nmol/L, 95% CI: 0.02 to 0.18, P=0.009). Moreover, a significant decrease in C-peptide values occurred in women with T2DM (WMD: -0.25 nmol/L, 95% CI: -0.30 to -0.20, P<0.001) versus healthy women (WMD: 0.02 nmol/L, 95% CI: -0.10 to 0.15, P=0.71) (**Supplementary Figure 1**).

### Publication bias and sensitivity analysis

The funnel plot of the effect sizes was essentially symmetrical and the Egger’s test confirm it (**Supplementary Figure 2**). These results remained essentially unchanged in the sensitivity analyses after we removed each RCT and combined the remaining ones (**Supplementary Figure 3**).

## Discussion

In this systematic review and meta-analysis, we examined the effect of the co-administration of 17β-estradiol and norethisterone acetate on markers of glucose and insulin metabolism, namely glycated haemoglobin (HbA1c), fasting glucose, insulin and C-peptide concentrations, in women. Based on data derived from 14 RCTs, our results suggest that the use of 17β-estradiol and norethisterone acetate can reduce fasting glucose, HbA1c, insulin and C-peptide levels in females. In the subgroup analyses, the age and BMI of the participants, the intervention length and the presence of type 2 diabetes mellitus (T2DM) seemed to mediate the impact of this drug combination on the examined markers of glucose and insulin metabolism.

Our findings clearly point out that the co-administration of 17β-estradiol and norethisterone acetate reduces HbA1c concentrations in females. The reduction was clinical significant (WMD: -0.65%, P=0.01) and was more notable when this drug combination was administered in elderly women (WMD: -0.62%, P<0.001 for females aged at least 60 years or more), in women diagnosed with obesity (WMD: -1.04%, P=0.03 for BMI ≥30 kg/m^2^) or T2DM (WMD: -0.84%, P=0.01), and when the length of the intervention was equal to or exceeded 6 months (WMD: -0.52%, P<0.001). This discovery is of particular importance as it has been revealed that HbA1c concentrations increase in women with advancing age. In a cohort study which enrolled nearly 170000 adults, Alghamdi et al. (2021) reported that HbA1c concentrations increase overall by 0.35% for each ten years increase in age, however, after the age of 50, the elevation in HbA1c is of 1.118% (32). Our results were also confirmed by other cohort studies. For example, Ferrara et al. demonstrated that the use of hormone replacement therapy (HRT) is linked with a reduction in HbA1c concentrations in women suffering from T2DM (P<0.001) (33). These conclusions were drawn based on a sub-analysis of the The Northern California Kaiser Permanente Diabetes Registry which recruited over 15000 females diagnosed with T2DM (33). Similarly, Kuh et al. depicted a positive association between HbA1c levels and age and communicated that women who were prescribed hormone replacement therapy (HRT) exhibited lower HbA1c, BMI and low-density lipoprotein cholesterol values (34). Likewise, in previous publications, we have pointed out that the co-administration of 17β-estradiol and norethisterone acetate reduces serum lipids’ concentrations, enabling thus a better metabolic control of T2DM in women (35, 36).

In terms of its impact on glycemia, the combined use of 17β-estradiol and norethisterone acetate was also successful in decreasing fasting glucose levels (WMD: -11.04 mg/dL, P<0.001). Similarly to the previously mentioned results, this type of hormone replacement therapy reduced fasting glucose when the duration of the administration was equal to or exceeded 6 months (WMD: -5.11 mg/dL, P=0.02) and when it was prescribed in females with concurrent T2DM (WMD: -29.20 mg/dL, P=0.01). However, fasting glucose values also declined in women who were at least overweight (WMD: -12.32 mg/dL, P<0.001 for BMI ≥25 kg/m^2^) or aged <60 years (WMD: -8.43 mg/dL, P<0.001). Taken as a whole, these findings reinforce the statement that the combination of 17β-estradiol and norethisterone acetate can be helpful in the management of T2DM in women as it improves glycemic control and is associated with a significant decrease in HbA1c and fasting glucose concentrations. This is particularly important in the prevention of T2DM-related complications, e.g., cardiovascular disease (CVD), as fasting glucose values have been linked to the 10-year risk of CVD in both pre- and postmenopausal women (37). What is more, the clinical guide for the management of T2DM during the menopause issued by the European Menopause and Andropause Society (EMAS) recommended a tailored-based approach in the prescription of HRT based on CVD risk: oral estrogens should be given to females with low risk of CVD, whereas transdermal estrogens should be administered in those with obesity or other risk factors for CVD. In addition to the use of estrogens, EMAS recommends supplementing the HRT regimen with a progestogen that does not influence carbohydrate metabolism, e.g., norethisterone (38). Moreover, the BMI seems to mediate the relationship between age and fasting glucose values in females, especially in those in whom menopause occurred later in life, e.g., after 54 years of age. Zhao et al. highlighted that the age at menopause was associated with the development of T2DM, overweight/obesity and fasting glucose concentrations (39). This information was confirmed by other investigations (40, 41). Even if these actions on glucose metabolism sound promising, we still need more RCT-based evidence to use HRT in the control or prevention of T2DM (42).

Insulin levels were also affected by the combined use of 17β-estradiol and norethisterone acetate. Our results suggest that, following the use of this drug combination, females exhibit a decline in insulin concentrations (WMD: -1.34 mIU/L, P=0.001). The decrease in insulin values was more notable in elderly women (WMD: -1.62 mIU/L, P=0.001 for age ≥60 years), women suffering from T2DM (WMD: -2.22 mIU/L, P<0.001) or overweight/obesity (WMD: -1.36 mIU/L, P=0.002) or when the length of the intervention exceeded or was equal to 6 months (WMD: -1.21 mIU/L, P=0.005). However, C-peptide concentrations only decreased in females diagnosed with overweight/obesity (WMD: -0.20 nmol/L, P<0.001 for BMI ≥25 kg/m^2^) or T2DM (WMD: -0.25 nmol/L, P<0.001). C-peptide concentrations are employed in clinical practice to evaluate the function of the beta cells of the pancreas, as this substance is generated in equimolar quantities to insulin as thus can reflect cardiometabolic health (43). These findings are in line with the results of previously published investigations which confirmed that HRT reduces fasting glucose, insulin and C-peptide values (44). In addition, Palla et al. pointed out that anthropometric indices indicative of an increased BMI, e.g., body perimeter and/or waist-to-hip ratio, are correlated with insulin and C-peptide levels, particularly in females with excessive body weight and other cardiometabolic ailments, e.g., metabolic syndrome (45). Thus, these women might be more sensitive to the action of 17β-estradiol and norethisterone acetate due to their excessive concentrations of insulin and C-peptide. It seems that 17β-estradiol can influence the levels of pro- and anti-inflammatory cytokines during the menopause (46). An assessment in a murine model reported that IL-10 concentrations increased, whereas IL-1β and TNF-α decreased, following the administration of this drug in aged rat hearts. Consequently, this compound was able to decrease insulin resistance and enhance insulin signalling which might explain the results of our meta-analysis (46).

Our study had several strengths and limitations. To our knowledge, this is the first meta-analysis to examine the impact of 17β-estradiol and norethisterone acetate on glucose metabolism in females. As the data was derived strictly from RCTs and the reported data is robust. In addition, we examined the inter-study heterogeneity and performed subgroup analyses based on several factors that could have influenced our findings, e.g., length of the intervention, age or BMI of the participants, presence of T2DM.

Several limitations must be also noted. The studies were conducted in multiple countries throughout the globe and thus the subjects had different demographics, clinical findings and most likely genetics. In addition, different brands of HRT drugs might have been administered based on the local availability. The studies conducted by Darko et al. (15) and Kernohan et al. (13) have wide confidence intervals due to their sample sizes, thus, their results do not provide a precise representation of the population mean. In addition, we combined just six studies that have selected C-peptide as an outcome variable and the findings of these investigations display significant differences.

Moreover, the inter-RCT heterogeneity was sometimes high and potential confounders apart from the ones explored in the subgroup analyses might have influenced the final results. The present study is the first systematic review and meta-analysis of randomized controlled trials (RCTs) which investigated the effects of 17β-estradiol plus norethisterone acetate treatment on fasting glucose, fasting insulin, glycated haemoglobin (HbA1c) and C-peptide concentrations in women based on articles published till June 14th, 2022. Future studies need to confirm the potential benefits of this drug combination in the prevention and/or management of cardiometabolic disorders.

### Clinical practice

The significant change in fasting glucose, HbA1c, insulin and C-peptide values by 17beta-estradiol plus norethisterone acetate reverses diabetes, obesity, and cardiometabolic disorders.

## Conclusion

Evidence to date highlights that the co-administration of 17β-estradiol and norethisterone acetate in females reduces fasting glucose, HbA1c, insulin and, in some instances, C-peptide concentrations.

## Data availability statement

The original contributions presented in the study are included in the article/**Supplementary Material**. Further inquiries can be directed to the corresponding author.

## Author contributions

WC and LZ carried out the concept, design and drafting of this study. WC and LZ reviewed literature, searched databases, screened articles, and extracted data. WC and LZ performed the acquisition, analysis, interpretation of data, and revision. All authors reviewed the manuscript for editorial and intellectual contents. All authors approved the final version of the manuscript.
