# Supplementary material for: The influence of 17β-estradiol plus norethisterone acetate treatment on markers of glucose and insulin metabolism in women: a systematic review and meta-analysis of randomized controlled trials
Source: Front Endocrinol (Lausanne). 2023 May 17;14:1137406. doi: 10.3389/fendo.2023.1137406 (PMC10230087; doi:10.3389/fendo.2023.1137406)
Supplement: Supplementary file 1 [file DataSheet_1.docx]

**Supplementary Table 1:** Quality assessment of trials included in this meta-analysis

|  | Random Sequence Generation (selection bias) | Allocation concealment (selection bias) | Blinding of participants and personnel (performance bias) | Blinding of outcome assessment (detection bias) | Incomplete outcome data (attrition bias) | Selective reporting (reporting bias) | Other bias | AHRQ standards |
| --- | --- | --- | --- | --- | --- | --- | --- | --- |
| Manassiev, N. | 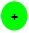 | 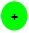 | 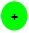 | 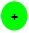 | 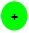 | 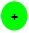 | 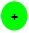 | Good |
| Fernandes, C. E. |  | 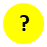 |  |  |  | 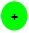 |  | Fair |
| Kernohan, A. F. | 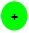 | 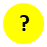 | 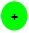 | 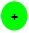 | 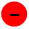 | 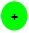 | 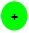 | Poor |
| Thunell, L. | 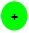 | 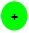 | 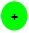 | 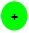 | 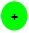 | 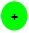 | 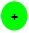 | Good |
| Osmanagaoglu, M. A. |  |  |  |  |  |  |  | Good |
| McKenzie, J. | 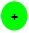 | 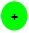 | 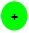 | 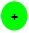 | 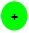 | 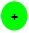 | 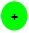 | Good |
| Samsioe, G. | 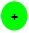 | 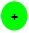 | 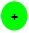 | 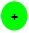 | 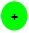 | 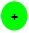 | 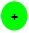 | Good |
| Samsioe, G. |  |  |  |  |  |  |  | Good |
| Walker, R. J. |  |  |  |  |  |  |  | Good |
| Ventura, P. |  |  |  |  |  |  |  | Good |
| Darko, D. A. |  | 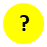 |  |  | 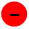 |  |  | Poor |
| Seed, M. |  |  |  |  | 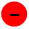 |  |  | Poor |
| Kimmerle, R.(a) |  |  |  |  |  |  |  | Good |
| Kimmerle, R.(b) |  |  |  |  |  |  |  | Good |
| Andersson, B. |  | 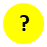 | 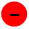 |  |  |  |  | Poor |
| Loke, D. F. M. |  | 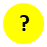 |  |  |  | 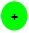 |  | Fair |
