# Supplementary material for: The influence of 17β-estradiol plus norethisterone acetate treatment on markers of glucose and insulin metabolism in women: a systematic review and meta-analysis of randomized controlled trials
Source: Front Endocrinol (Lausanne). 2023 May 17;14:1137406. doi: 10.3389/fendo.2023.1137406 (PMC10230087; doi:10.3389/fendo.2023.1137406)
Supplement: Supplementary file 3 [file DataSheet_3.docx]

A) HbA1c (P = 0.941) B) FBS (P= 0.245)

C) Insulin (p = 0.054) D) C-peptide (p = 0.572)

**Supplementary Figure 2.** Funnel plot of the weighted mean difference (WMD) versus the s.e. of the weighted mean difference (WMD).
