# Supplementary material for: The influence of 17β-estradiol plus norethisterone acetate treatment on markers of glucose and insulin metabolism in women: a systematic review and meta-analysis of randomized controlled trials
Source: Front Endocrinol (Lausanne). 2023 May 17;14:1137406. doi: 10.3389/fendo.2023.1137406 (PMC10230087; doi:10.3389/fendo.2023.1137406)
Supplement: Supplementary file 4 [file DataSheet_4.docx]

**Supplementary Figure 3.** Sensitivity analysis.

A) HbA1c

B) FBS

C) Insulin

D) **C-peptide**
